# Impact of Coronavirus Diseases on Liver Enzymes

**DOI:** 10.7759/cureus.17650

**Published:** 2021-09-01

**Authors:** Ammara Naeem, Manoj Kumar Khamuani, Pardeep Kumar, FNU Pooja, Deepak Raj, Kirshan Lal, Wajeeha Shahid, Waseem Mahar, Amber Rizwan, Aliya Fatima

**Affiliations:** 1 Internal Medicine, Lahore General Hospital, Lahore, PAK; 2 Internal Medicine, Liaquat University of Medical and Health Sciences, Jamshoro, PAK; 3 Internal Medicine, Chandka Medical College, Larkana, PAK; 4 Internal Medicine, Ghulam Muhammad Mahar Medical College, Sukkur, PAK; 5 Internal Medicine, Jinnah Sindh Medical University, Karachi, PAK; 6 Internal Medicine, Ghulam Muhammad Mahar Medical College, Karachi, PAK; 7 Family Medicine, Jinnah Postgraduate Medical Centre, Karachi, PAK; 8 Internal Medicine, Jinnah Postgraduate Medical Centre, Karachi, PAK

**Keywords:** covid-19, novel corona virus, liver enzymes, hepatic enzymes, sars-cov-2 (severe acute respiratory syndrome coronavirus -2)

## Abstract

Introduction

Coronavirus disease 2019 (COVID-19) affects various organs including lungs, brain, and eyes. Very limited data is available related to the effect of COVID-19 on liver. This study is conducted to determine the impact of COVID-10 on liver by measuring the frequency of participants with deranged liver enzymes in patients diagnosed with COVID-19.

Methods

This cross-sectional study was conducted in a COVID-19 unit of a tertiary care hospital in Pakistan from February 2021 to June 2021. A total of 900 patients admitted with COVID-19 were enrolled in the study after seeking informed consent. After enrollment, taking history and vitals, 5 mL blood was drawn via phlebotomy and sent to the laboratory to test for C-reactive protein, lactate dehydrogenase, and liver enzymes.

Results

Overall 141 (28.2%) participants had a minimum of one deranged liver enzyme. The most commonly deranged liver enzyme found was alanine transaminase (ALT), both in males (19.9%) and females (21.3%), followed by aspartate transaminase (male: 18.3% and female: 20.3%). Serum total bilirubin was deranged in both males (8.4%) and females (8.3%). There was no significant difference in the gender-wise prevalence of deranged liver enzymes.

Conclusion

Liver enzymes are frequently deranged in patients admitted with COVID-19. Liver enzymes should be regularly monitored during the course of management of COVID-19, as various medications used in the treatment of COVID-19 may further deteriorate liver enzymes and may cause long-term damage.

## Introduction

Around the globe, healthcare systems have faced immense challenges due to the coronavirus disease 2019 (COVID-19) pandemic [1]. Severe acute respiratory syndrome coronavirus 2 (SARS-CoV-2) belongs to the Coronaviridae family of the virus and betacoronavirus genus. SARS-CoV-2 and the Middle East respiratory syndrome-related coronavirus (MERS-CoV) also belong to the family Coronaviridae [2].

Fever, cough, dyspnea, and chest pain are the symptoms commonly reported in cases of COVID-19. Ground-glass opacities are seen on radiological investigations, suggesting pneumonia [3]. Other than conventional symptoms, many other organs are affected as well by COVID-19. The frequency of neurological symptoms in COVID-19 cases is appreciable. Mostly the neurological symptoms reported are dizziness, headache, impairment of taste and smell [4]. In patients with COVID-19, ophthalmological symptoms were also found. The most common of those symptoms is conjunctival irritation, followed by diplopia and cotton wool spots [5]. The long-term impact of COVID-19 has been identified. Top five symptoms reported are fatigue (58%), headache (44%), attention disorder (27%), hair loss (25%), and dyspnea (24%) [6].

The impact of COVID-19 on the function of the liver is under-studied and there is insufficient data regarding the impact of COVID-19 on liver enzymes. Therefore, this study is conducted to determine the presence of deranged liver enzymes in patients diagnosed with COVID-19.

## Materials and methods

This cross-sectional study was conducted in a COVID-19 unit of a tertiary care hospital in Pakistan from February 2021 to June 2021. Total 900 patients, admitted with COVID-19, were enrolled in the study after seeking informed consent. Participants were enrolled via consecutive convenient non-probability sampling. Participants were explained about research protocol and any queries were addressed. Ethical review board approval was taken from Liaquat University of Medical Health Sciences (LUMHS/2021/COVID/IRB-04) before the enrollment of patients. Sample for COVID-19 was taken via nasal swap. COVID-19 was diagnosed by polymerase chain reaction. Patients with a history of liver disease, recent hepatitis, gallstones, or on drugs that affect liver enzymes were excluded from the study.

After enrollment, the patient’s vitals such as respiratory rate and oxygen saturation were noted in the questionnaire, and 5 mL blood was drawn via phlebotomy and sent to the same laboratory to test for C-reactive protein (CRP), lactate dehydrogenase (LDH), and liver enzymes. All tests were conducted from the same laboratory to maintain consistency. 

SPSS Statistics, v. 23.0 (IBM Corporation, Armonk, New York, United States) was used to analyze the collected data. For numerical data, mean and standard deviation (SD) were calculated, while frequency and percentage were calculated for categorical data. Independent t-test and chi-square were used when deemed appropriate. A p-value of less than 0.05 indicated a statistically significant difference between the two groups and the null hypothesis is void.

## Results

The mean age of participants at the time of admission was 46 ± 07 years. There were 261 (52.2%) males and 239 (47.8%) females. CRP value was 110.7 ± 14.3 mg/dL and mean SaO2 was 88.2 ± 4.3% at the time of admission. Other demographics are defined in Table 1.

**Table 1:** Characteristics of study participants BPM: breaths per minute, CRP: C-reactive protein, °F: Fahrenheit, IU: international unit, LDH: lactate dehydrogenase, mg/dL: milligram per decilitre, SaO2: Oxygen saturation

Characteristics at the time of admission	Value
Age (in years)	46 ± 07
Male	261 (52.2%)
Temperature (°F)	100.6 ± 0.7
Respiratory Rate (BPM)	30.6 ± 4.9
CRP (mg/dL)	110.7 ± 14.3
LDH (IU)	325.6 ± 87.5
SaO_2_ (%)	88.2 ± 4.3

The mean alanine transferase (ALT) values in males and females were 62.51 ± 32.01 IU and 42.14 ± 15.14 IU, respectively. The mean gamma-glutamyl transferase (GGT) value in males was 52.21 ± 28.21 IU and 45.26 ± 19.72 IU in females. Serum total bilirubin in males was 12.14 ± 3.59 umol/L and in females, it was 13.17 ± 3.59 umol/L (Table 2).

**Table 2:** Genderwise comparison of mean liver enzyme values ALT: alanine aminotransferase, AST: aspartate aminotransferase, GGT: gamma-glutamyl transferase, IU: international unit, umol/L: micromole per liter

Mean liver enzyme values	Normal Laboratory Range	Our Study	
		Male (n=261)	Female (n=239)
Serum total bilirubin (umol/L)	1.71-20.52	12.14 ± 3.59	13.17 ± 3.59
Serum direct bilirubin (umol/L)	0-3.42	1.54 ± 0.34	1.54 ± 0.17
Serum indirect bilirubin (umol/L)	1.71-13.68	10.62 ± 2.05	10.09 ± 1.88
Serum GGT (IU)	Male: < 55, Female: < 38	52.21 ± 28.21	45.26 ± 19.72
ALT (IU)	Male: < 45, Female: < 35	62.51 ± 32.01	42.14 ± 15.14
AST (IU)	Male: < 35, Female: < 31	35.02 ± 14.51	30.82 ± 13.21

Overall 141 (28.2%) participants have a minimum of one deranged liver enzyme. The most commonly deranged liver enzyme was ALT, both in males (19.9%) and females (21.3%), followed by AST (male: 18.3% and female: 20.3%). Serum total bilirubin was deranged in 8.4% of male participants and 8.3% of the female participants. There was no significant difference in the prevalence of deranged liver enzymes between males and females (Table 3).

**Table 3:** Prevalence of deranged liver enzymes ALT: alanine aminotransferase, AST: aspartate aminotransferase, GGT: gamma-glutamyl transferase, IU: international unit, mg/dL: milligrams per decilitre, NS: nonsignificant

Prevalence of deranged liver enzymes	Male (n=261)	Female (n=239)	p-value
Serum total bilirubin (mg/dL)	22 (8.4%)	20 (8.3%)	NS
Serum direct bilirubin (mg/dL)	26 (9.9%)	22 (9.2%)	NS
Serum indirect bilirubin (mg/dL)	14 (5.3%)	12 (5.0%)	NS
Serum GGT (IU)	43 (16.4%)	38 (15.9%)	NS
ALT(IU)	52 (19.9%)	51 (21.3%)	NS
AST (IU)	48 (18.3%)	48 (20.3%)	NS

## Discussion

Our study demonstrated that 28.2% of participants had a minimum of one deranged liver enzyme. However, the most common of the deranged liver enzymes was ALT, followed by AST and serum GGT in both genders. Moreover, serum total bilirubin was almost equally deranged in both genders, i.e. 8.4% in males and 8.3% in females. Another important finding of our study was that the mean range of ALT, AST, and serum GGT was higher than the normal values.

Supporting the results of our study, the literature provides some large-scale studies that assessed the clinical features of COVID-19 patients and some factors that could potentially lead to liver injury caused by COVID-19. In these studies, higher levels of ALT and AST were observed [7-10]. Several case reports have also shown abnormal liver function tests (LFTs) in patients with COVID-19 [7,11,12]. In a study, patients grouped based on the severity of the disease indicated that severe COVID-19 patients had higher levels of ALT and AST compared to those with mild disease [9,13,14].

The theory that might explain the effect of COVID-19 on liver is related to presence of angiotensin-converting enzyme 2 receptors. As angiotensin-converting enzyme 2 receptors are expressed on cholangiocytes, COVID-19 can conveniently attack the liver. While examining the liver of COVID-19 patients, endothelitis was noticed [15], and liver sinusoids showed fibrin microthrombi [16]. When 48 cases of liver biopsies were studied, liver sinusoids demonstrated dilation of portal vein branches, luminal thrombosis, portal tract fibrosis, and microthrombi in the sinusoids [17].

In view of the above-mentioned literature, higher levels of liver enzymes are observed in critical COVID-19 patients. Cai et al. stated that with the administration of lopinavir-ritonavir, changes in LFTs are expected to increase seven times [18]. Similarly, a clinical trial conducted on 199 patients with severe COVID-19 demonstrated that increases in ALT, AST, and total bilirubin levels were more often observed in the lopinavir-ritonavir group compared to those who did not receive this. Remdesivir has also been reported to cause early recovery of COVID-19 patients [19]. In a trial assessing Remdesivir treatment for five or 10 days, a considerable increase in ALT/AST levels was observed in 4-6% of patients as well as a life-threatening increase in 2-3% of patients, leading to the discontinuation of the treatment [20]. Acetaminophen, a drug used to treat COVID-19 symptoms, is known to cause fluctuations in aminotransferases even at normal doses [21]. Previous data is not available on the relation between hydroxychloroquine and its effect on LFTs [22,23].

This study points towards the idea that COVID-19 patients should be screened for liver enzymes at regular intervals for better management of the disease. However, since it is a single-center study, future studies involving multiple centers with a larger sample size are required to confirm the findings of our study.

## Conclusions

Liver enzymes are frequently deranged in patients admitted with COVID-19. Liver enzymes should be regularly monitored during the course of management of COVID-19, as various medications used in the treatment of COVID-19 may further deteriorate liver enzymes and may cause long-term damage. Moreover, proper screening of liver enzymes would also help in better treatment of patients with underlying liver conditions to minimize the chances of further complications.
